# SARS-CoV-2 Reinfection in a Healthcare Worker Despite the Presence of Detectable Neutralizing Antibodies

**DOI:** 10.3390/v13040661

**Published:** 2021-04-12

**Authors:** Thomas Theo Brehm, Susanne Pfefferle, Ronald von Possel, Robin Kobbe, Dominik Nörz, Stefan Schmiedel, Adam Grundhoff, Flaminia Olearo, Petra Emmerich, Alexis Robitaille, Thomas Günther, Platon Braun, Gabriele Andersen, Johannes K. Knobloch, Marylyn M. Addo, Ansgar W. Lohse, Martin Aepfelbacher, Nicole Fischer, Julian Schulze zur Wiesch, Marc Lütgehetmann

**Affiliations:** 1 I.Department of Internal Medicine, University Medical Center Hamburg-Eppendorf, Martinistraße 52, 20246 Hamburg, Germany; t.brehm@uke.de (T.T.B.); r.kobbe@uke.de (R.K.); s.schmiedel@uke.de (S.S.); m.addo@uke.de (M.M.A.); a.lohse@uke.de (A.W.L.); j.schulze-zur-wiesch@uke.de (J.S.z.W.); 2 German Center for Infection Research (DZIF), Partner Site Hamburg-Lübeck-Borstel-Riems, Germany; s.pfefferle@uke.de (S.P.); adam.grundhoff@leibniz-hpi.de (A.G.); j.knobloch@uke.de (J.K.K.); 3 Institute of Medical Microbiology, Virology and Hygiene, University Medical Center Hamburg-Eppendorf, Martinistraße 52, 20246 Hamburg, Germany; d.noerz@uke.de (D.N.); f.olearo@uke.de (F.O.); m.aepfelbacher@uke.de (M.A.); nfischer@uke.de (N.F.); 4 Bernhard Nocht Institute for Tropical Medicine, Bernhard-Nocht-Straße 74, 20359 Hamburg, Germany; vonpossel@bnitm.de (R.v.P.); emmerich@bnitm.de (P.E.); 5 Department of Tropical Medicine and Infectious Diseases, Center of Internal Medicine II, University of Rostock, 18051 Rostock, Germany; 6 Heinrich Pette Institute, Leibniz Institute for Experimental Virology, Martinistraße 52, 20251 Hamburg, Germany; alexis.robitaille@leibniz-hpi.de (A.R.); thomas.guenther@leibniz-hpi.de (T.G.); 7 Staff and Faculty Health Services, University Medical Center Hamburg-Eppendorf, Martinistraße 52, 20246 Hamburg, Germany; p.braun@uke.de (P.B.); gabriele.andersen@uke.de (G.A.)

**Keywords:** SARS-CoV-2, reinfection, COVID-19, healthcare worker, immunity, neutralizing antibodies

## Abstract

So far, only a few reports about reinfections with SARS-CoV-2 have been published, and they often lack detailed immunological and virological data. We report about a SARS-CoV-2 reinfection with a genetically distinct SARS-CoV-2 variant in an immunocompetent female healthcare worker that has led to a mild disease course. No obvious viral escape mutations were observed in the second virus variant. The infectious virus was shed from the patient during the second infection episode despite the presence of neutralizing antibodies in her blood. Our data indicate that a moderate immune response after the first infection, but not a viral escape, did allow for reinfection and live virus shedding.

## 1. Introduction

Severe acute respiratory syndrome coronavirus 2 (SARS-CoV-2) has infected more than 135 million people and caused more than 3 million deaths worldwide until now [1]. Infection of the immunocompetent host normally leads to the development of neutralizing antibodies, yet antibody levels may wane over time [2]. Reports of reinfections have been anecdotally published with increasing frequency [3,4,5,6,7,8,9,10]. A more profound understanding of the virological and immunological characteristics of SARS-CoV-2 reinfections may help to define reliable correlates of immunity. Here, we present detailed clinical, virological, and immunological data of the first well-documented case of a SARS-CoV-2 reinfection in a highly exposed immunocompetent female healthcare worker in Germany, which occurred seven months after her initial infection. Importantly, all criteria suggested by the recently published Centers for Disease Control and Prevention (CDC) protocol for investigating suspected SARS-CoV-2 reinfection were met (duration since previous test > 90 days, CT value < 33, symptoms typical of coronavirus disease 2019 (COVID-19), observation of different clades between the first and second infection) [10]. Successful SARS-CoV-2 isolation in cell culture at the time of reinfection proves that shedding of the infectious virus was possible despite the presence of preformed neutralizing antibodies.

## 2. Materials and Methods

### 2.1. Quantitative Real-Time Polymerase Chain Reaction (qRT-PCR)

For detection and quantification of SARS-CoV-2 RNA, the fully automated qRT-PCR system Cobas6800 (Roche Molecular Solutions, Pleasanton, CA, USA) was used. The viral load was calculated using the E-gene target (T2) and a standard curve to convert from Ct to viral loads using a commercial quantitative reference standard (from Instand, Düsseldorf, Germany). The linear range and matrix validation have previously been published by our group [11].

### 2.2. Cell Culture and Virus Isolation

For virus isolation, 500 µL of the swab specimen taken at the time of reinfection (29 December 2020) was used to infect Vero E6 cells (ATCC CRL-1008) [12]. Virus growth was confirmed by qRT-PCR at 72 h post-infection. The supernatant was filtered and transferred to fresh Vero cells. After two days, cells showed a strong cytopathogenic effect (CPE) and the supernatant was harvested and frozen. The median tissue culture infectious dose (TCID50) was calculated based on the infection of Vero cells with serial ten-fold dilutions of the stock and was 1.57 × 10^7^/mL.

### 2.3. Viral Whole Genome Sequence Analysis

The viral genomes from the first and second episode of infection were sequenced from the pharyngeal swab material and were named #HH-24.I and HH-24.II, respectively. Amplicon sequencing and a bioinformatic analysis were performed, as recently published [13,14]. Library generation was performed using the CleanPlex SARS-CoV-2 Panel (Paragon Genomics, CA, USA). Merged reads were aligned to NC_045512.2 using minimap2 [15] with default settings for short read alignment. Major variants (≥50% of reads) were called using freebayes Bayesian haplotype caller v1.3.1 [16] with ploidy and haplotype independent detection parameters to generate frequency-based calls for all variants passing input thresholds (-K -F 0.5). Input thresholds were set to a minimum coverage of 10 and minimum base quality of 30 (min-coverage 10, -q30). Resulting variants were annotated using ANNOVAR [17]. Pangolin lineage and nextstrain clade assignment of consensus sequences were performed using the pangolin (https://github.com/cov-lineages/pangolin, accessed on 7 March 2021) and nextclade (https://github.com/nextstrain/nextclade, accessed on 7 March 2021) packages. Phylogenetic analysis and tree visualization were performed using nextstrain [18]. To visualize the investigated samples in the context of European SARS-CoV-2 strains, 100 European sequences were randomly sub-sampled from the data available in the GISAID database [19]. For more detailed methods of phylogenetic analyses see [13,14].

### 2.4. Analysis of Humoral Immune Response

An automated quantitative anti-SARS-CoV-2 IgG assay targeting the S1/S2 spike domain (DiaSorin, Saluggia, Italy) was used according to the manufacturer’s recommendations [20]. For the immunofluorescence assay, Vero E6 cells (ATCC CRL-1008) infected with SARS-CoV-2 isolate HH-1 were spotted on glass slides, air-dried, and fixed in ice-cold acetone. Serial dilutions of patient sera were incubated on slides for 1 h at 37 °C. The slides were washed twice with PBS. SARS-CoV-2 antibodies (IgM, IgG, IgA) were detected by indirect immunofluorescence using anti-human IgG (Medac, Cat. No 5230–0288), anti-human IgM (Medac, Cat. No 02-10-03) and anti-human IgA (ThermoFisher, Waltham, MA, USA, Cat. No. A18782) (FITC labeled secondary antibodies and incubation at 37 °C for one hour).

### 2.5. Virus Neutralization Assay (NT)

Patient sera drawn at different timepoints before and after the reinfection were available for virus neutralization assays. The sera used were decomplemented at 56 °C for 30 min prior to serial dilution in triplicate starting at 1:20. Triplicates of the dilutions were mixed with an equal volume of SARS-CoV-2 (isolate HH-1/isolate HH-24.2), equivalent to 20 TCID50 isolate HH-1/4 TCID50 isolate HH-24.2 per sample. After incubation at 37 °C for one hour, the serum/virus mixtures were transferred to 96-well plates containing 5.0 × 10^6^ cells/plate of Vero cells (ATCC CRL-1008) seeded the previous day. Following incubation for 96 h at 37 °C, supernatants were discarded, and the plates were fixed in 4% formaldehyde and stained with crystal violet. The highest serum dilution protecting 2 of 3 wells from cytopathic effect (CPE) was taken as the neutralizing antibody titer.

## 3. Results

**Figure 1:**
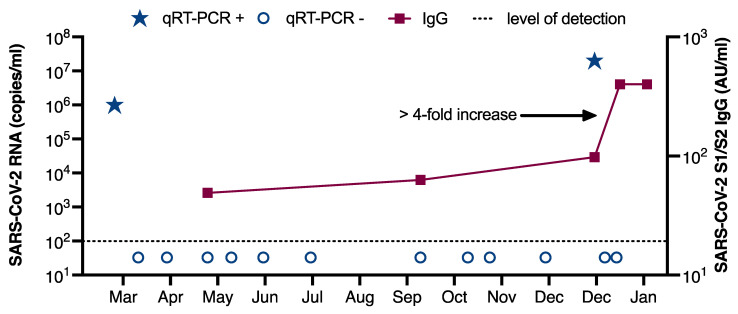
Time course with quantitative detection of SARS CoV-2 RNA [log copies/mL] (blue) and quantitative anti SARS CoV-2 S1/S2 antibody levels [log AU/mL] (red). RNA level was 1 × 10^6^ copies/mL and 2 × 10^7^ copies/mL at first infection and reinfection respectively. Anti-SARS-CoV-2 spike (S1/S2) IgG was 40 IU/mL after first infection and a > 4-fold booster during reinfection was observed (97 AU/mL on 29 December 2020, and >400 AU/mL on 13 January 2021).

A 27-year-old female nurse working in a COVID-19 ward of the University Medical Center Hamburg-Eppendorf (UKE), Germany, developed fever, chills, and exertional dyspnea on 18 March 2020. She had no history of any underlying medical conditions and no indication of a compromised immunity. The patient immediately placed herself into self-quarantine and was tested positive for SARS-CoV-2 on March 20 by qRT-PCR of a naso- and oropharyngeal swab with 1 × 10^6^ copies/mL (Figure 1) [21]. While the fever and chills resolved during domestic isolation on March 25, she reported exertional dyspnea for another four weeks. Notably, mild arterial hypertension was first diagnosed a few weeks after the infection, and treatment with bisoprolol was initiated. She returned to work at her ward after 17 days of quarantine, after testing negative for SARS-CoV-2 by qRT-PCR in two subsequent samples. Anti-SARS-CoV-2 spike (S1/S2) IgG levels in July 2020 were 40 AU/mL in July 2020 and thus clearly above the 15 AU/mL detection limit suggested by the manufacturer. At later time points, anti-spike (S1/S2) IgG levels remained stable (60 AU/mL in September 2020) (Figure 1 and Supplementary Materials Table S1). During the night shift of 26 to 27 December 2020, she developed a dry cough and mild rhinorrhea. Routine hospital surveillance by qRT-PCR (twice weekly using gargling solution) returned a positive result for SARS-CoV-2 on 27 December (<5000 copies/mL), 282 days after the first positive test. Consecutive qRT-PCR tests from naso- and oropharyngeal swabs on 28 and 29 December showed viral loads of 9 × 10^5^ and 2 × 10^7^ copies/mL, respectively.

**Figure 2:**
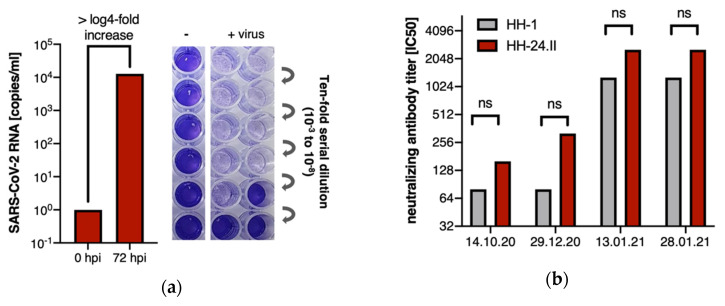
(**a**) Successful isolation of SARS-CoV-2 from swab sample (HH-24.II) reflected by > log4-fold in-crease of viral RNA in the supernatant of Vero cells at 72 h post infection (hpi) detected by qRT-PCR. Quantification of the virus stock produced of the rescued virus in cell culture revealed a TCID50 of 1.57 × 10^7^; (**b**) Virus neutralization assay was performed with serial dilutions of patient sera of one time point before (14 October 2020) and three time points after the reinfection (29 December 2020, 13 January 2021, 28 January 2021) and both the isolated virus of the patient (HH-24.II, red bars) and the HH-1 isolate (gray bars). Neutralizing antibody titers (IC50) were detected at all time points. No significant differences in the neutralizing capacity of the two linages were observed. Between 29 December 2020 and 13 January 2021 a > 4-fold titer increase was observed which reflects a significant increase.

At the time of reinfection, inflammatory parameters were not elevated and leukocyte subsets, as well as total IgM, IgG and IgA levels, were within normal range, without any indication of immunodeficiency (Supplementary Materials Table S2). Symptoms were resolved by 30 December 2020, and she was tested negative for SARS-CoV-2 on 11 January 2021. During the second infection, a rapid increase in the anti-SARS-CoV-2 spike (S1/S2) IgG was observed (97 AU/mL on 29 December 2020 and >400 AU/mL on 13 January 2021). Indirect immunofluorescence assays demonstrated a low IgM titer only at the first infection, and no measurable IgA titers, but significant IgG titer increases after the first and second infection (Supplementary Materials Table S1). The SARS-CoV-2 variant causing the reinfection was successfully isolated in cell culture (Figure 2a). No samples of the first infection were available for virus rescue attempts. Therefore, neutralizing antibody assays (NT IC50) were performed with both our local Hamburg reference isolate (HH-1) [12] and the virus isolated at the time of reinfection (HH-24.II) on patient sera drawn before and after the reinfection. We observed similar neutralizing titers with 1:80 and 1:160 after the first infection and strongly increased neutralizing titers of 1:1280 and 1:2560 after the second infection for both isolates HH-1 and HH-24.II, respectively (Figure 2b).

**Figure 3:**
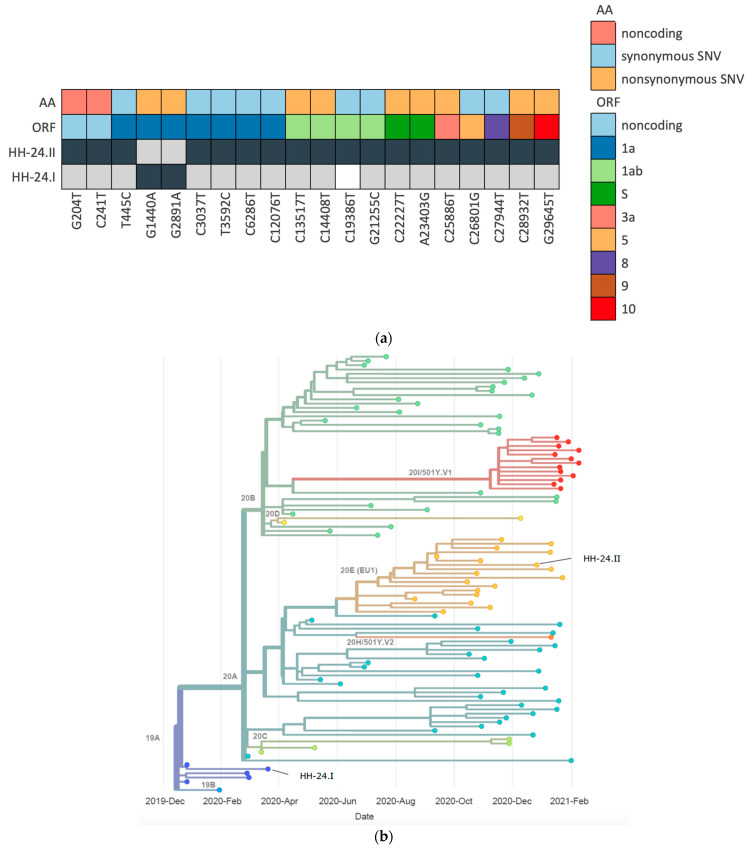
(**a**) Variant nucleotide positions of the sequences HH-24.I and HH-24.II with respect to the reference sequence NC_045512.2 are given in dark grey, whereas light grey boxes indicate reference bases. Uncovered positions by amplicon seq are depicted as white boxes. The color bars on top represents feature assignment (ORF, open reading frame; AA, amino acid changes); (**b**) nextstrain phylogenetic visualization of HH-24.I and HH-24.II in the context of 100 random samples present in Europe from onset of the pandemic until February 2021. Clades are indicated by different colors.

Next generation sequencing (NGS) revealed that the viral sequences from the initial virus variant in March (HH-24.I) and the variant in December (HH-24.II) belonged to pangoline lineages B.3 and B.1.177, respectively [22] (Figure 3b). In total, both sequences differ in 21 positions (Supplementary Materials Table S3 and Figure 3a), including two typical variations in spike proteins A222V and D614G.

## 4. Discussion

Homologous reinfections with seasonal human betacoronaviruses within one year have previously been reported and are generally associated with milder symptoms in reinfected patients compared to primary infections [23,24]. The previously reported reinfections with SARS-CoV-2 have been either entirely asymptomatic [3,9], less severe [5], like in our patient, or more severe [4,6,7,8] in relation to the initial episode. Since reinfection cases are usually noticed because of clinical symptoms, there is likely a reporting bias towards symptomatic cases and post-infection immunity to SARS-CoV-2 may generally protect from severe illness. This is supported by a recent study which demonstrated that seropositive healthcare workers had a substantially reduced risk of SARS-CoV-2 reinfection in the six months following the initial infection [25]. However, correlates of protection remain to be established and it is currently unknown whether the mode of reinfection (e.g., overwhelmingly high titers of SARS-CoV-2, virus-intrinsic virulence), the magnitude, breadth, and quality of the humoral immune responses, waning T-cell immunity or other viral or host factors alone or in combination allow for reinfections [26]. According to recently published case definitions for confirmed SARS-CoV-2 reinfections, viral RNA sequencing is required to differentiate a true reinfection by distinct viral variants from prolonged viral shedding or a reactivation of a lingering virus infection [10,27]. While it has been shown that in most patients, SARS-CoV-2 RNA is undetectable four weeks after the onset of symptoms, prolonged PCR-positivity of up to 104 days after the initial infection has been reported [28,29]. We demonstrate that the same person was infected with SARS-CoV-2 in March 2020 and was reinfected 282 days after the first positive qRT-PCR with a different viral lineage (19A and 20 EU1 respectively). The SARS-CoV-2 sequences retrieved from both infections matched the epidemiology of the typical clades circulating in Germany during the respective time without any additional changes in the essential parts of the spike gene associated with immune escape. Moderate levels of SARS-CoV-2 spike IgG and an NT (IC50) antibody assay were observed after the initial infection. Those were comparable to levels detectable after a mild clinical course of COVID-19 or two weeks after a single injection of an mRNA vaccine [30]. The current reinfection episode was associated with only a few symptoms and was detected only after a routine screening of exposed healthcare workers at our hospital [31]. It resulted in a strong (approximate factor of 10) boost of antibody levels in both the quantitative anti-spike (S1/S2) IgG and in a 4-fold titer increase in the NT (IC50) antibody assay. Notably, the peak virus titer at the time of reinfection was not reduced compared to the initial infection, but the virus was cleared rapidly and was below the limit of detection of diagnostic qRT-PCR after nine days. There is currently little knowledge about the level and quality of humoral immune responses that can render protection from clinical disease, and a better understanding about reinfection events may help to identify serological correlates of immunity. Further studies are needed to investigate whether vaccination after COVID-19 or reinfection reliably boosts the SARS-CoV-2-specific immune responses to levels where sterilizing immunity and longer lasting protection from clinical disease and transmission are achieved.

## 5. Conclusions

The presented case of a SARS-CoV-2 reinfection of an immunocompetent patient in a high-risk healthcare setting indicates that a moderate immune response after the first infection rather than viral escape did allow for the reinfection. The shedding of the infectious virus in the presence of neutralizing antibodies indicates that during reinfection, further transmission of SARS-CoV-2 is conceivable.

## Data Availability

No new data were created or analyzed in this study. Data sharing is not applicable to this article.

## Supplementary Materials

The following are available online at https://www.mdpi.com/article/10.3390/v13040661/s1, Table S1: Serological results, Table S2: Laboratory parameters at the time of reinfection, Table S3: Variant description.
