# Supplementary material for: SARS-CoV-2 Reinfection in a Healthcare Worker Despite the Presence of Detectable Neutralizing Antibodies
Source: Viruses. 2021 Apr 12;13(4):661. doi: 10.3390/v13040661 (PMC8070424; doi:10.3390/v13040661)
Supplement: Supplementary file 1 [file viruses-13-00661-s001.pdf]

Table S1. Serological results

Date	Indirect immunofluorescence assay [antibody titer]			Neutralisation assay [titer IC50]	
	**IgG**	**IgM**	**IgA**	**HH-1**	**HH-24.II**
02.07.20	640	80	<20	80	160
30.07.20	1280	<20	<20	160	160
14.10.20	640	<20	<20	80	160
29.12.20	320	<20	<20	80	320
13.01.21	2560	<20	<20	1280	2560
28.01.21	1280	<20	<20	1280	2560

Table S2. Laboratory parameters at the time of reinfection

Parameter	Normal range	Value
WBC (10^9/*L*)	3.8 – 11.0	3.6
Neutrophil count (10^9/*L*)	1.5 – 7.7	1.5
Lymphocytes (10^9/*L*)	1.0 – 3.6	1.5
T cells (/µL)	900 – 2900	1155
CD4+ cells (/µL)	500 – 1350	831
CD8+ cells (/µL)	290 – 930	268
CD4+/CD8+ ratio	0.6 – 3.6	3.1
B cells (/µL)	80 – 500	200
NK cells (/µL)	35 – 350	140
Platelets (10^9/*L*)	150 – 400	222
Hemoglobin (g/dL)	12.3 – 15.3	11.3
HbA1C (%)	4.8 – 5.6	5.7
C-reactive protein (mg/L)	<5	<4
Procalcitonin (µg/L)	<0.5	<0.02
Interleukin 6 (ng/L)	<4.4	<2.7
Ferritin (µg/L)	10.0 – 291.0	9.2
IgG (g/L)	6.5 – 16.0	16.0
IgA (g/L)	0.4 – 3.5	1.6
IgM (g/L)	0.5 – 3.0	0.6

Table S3. Variant description

	Variants	Amino acid substitution	Nextstrain clade	Pangolin lineage
**HH-24.I**	G1440A, G2891A	ORF1a: G392D<br>ORF1a: A876T	19A	B.3
**HH-24.II**	G204T, C241T, T445C, C3037T, T3592C, C6286T, C12076T, C13517T, C14408T, C19386T, G21255C, C22227T, A23403G, C25886T, C26801G, C27944T, C28932T, G29645T	ORF1b: T17I<br>ORF1b: P314L<br>S: A222V<br>S: D614G<br>ORF3a: S165F<br>ORF9(N): A220V<br>ORF10: V30L	20E (EU1)	B.1.177
